# Professor Alain Chabaud (13 March 1923–11 March 2013)

**DOI:** 10.1051/parasite/2014013

**Published:** 2014-04-24

**Authors:** Jean-Antoine Rioux, Marie-Claude Durette-Desset, Jean-Louis Albaret, Christiane Bayssade-Dufour, Jimmy Cassone, Irène Landau, Annie Petter, Roselyne Tchéprakoff

**Affiliations:** 1 Honorary Professor of Parasitology (Montpellier), Former President of the French Society of Parasitology, Faculté de Médecine, Université Montpellier 1 1 rue de l’École de Médecine 34000 Montpellier France; 2 Ex-members of “Laboratoire de Zoologie-Vers”, Muséum National d’Histoire Naturelle Paris France

## Career and Achievements

by Jean-Antoine Rioux

**Figure 1.**
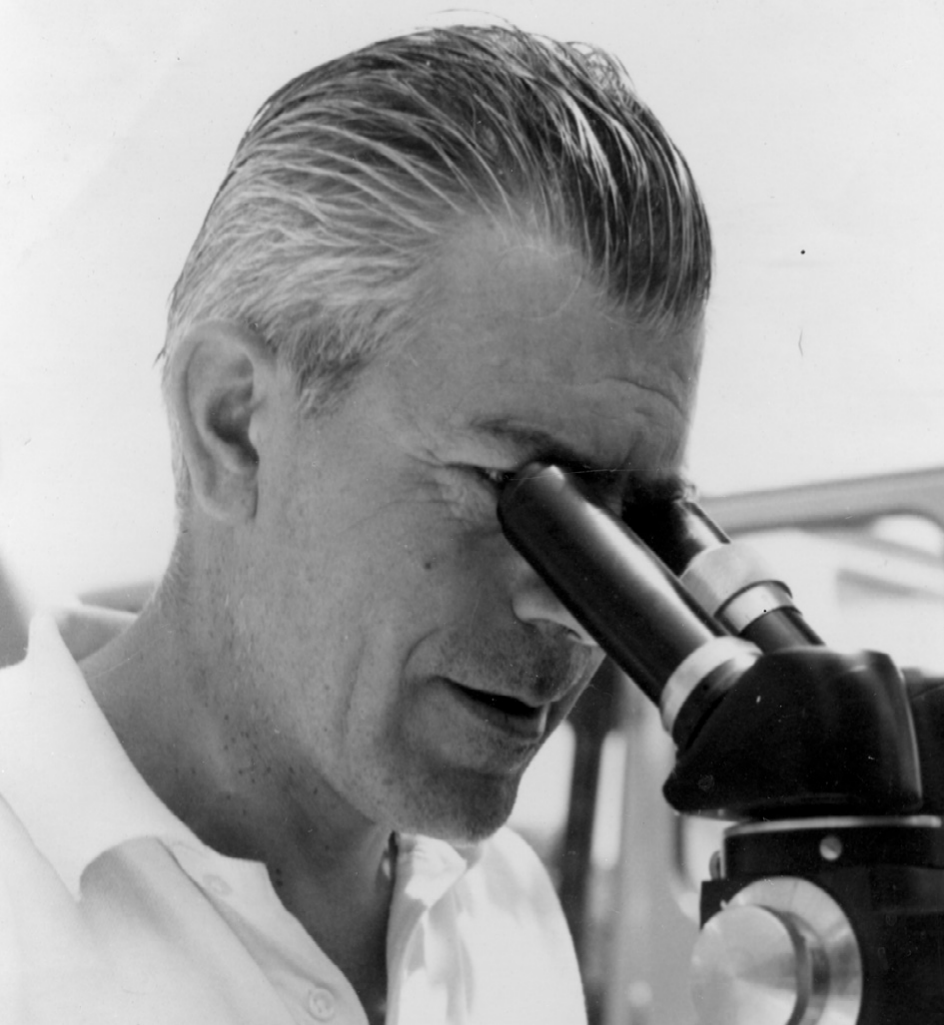
Professor Alain Chabaud (13 March 1923–11 March 2013).

### A grassroots naturalist

Alain Chabaud was raised in green Normandy, and very early became a full-fledged naturalist. He may have been born with this passion, or it could have been the result, as he liked to point out, of his contact with a nature-enthusiast primary school teacher who marked his early youth. This was a happy period when hedges and groves had not yet been decimated by reparcelling. This temperament prevailed throughout his long and successful career. Although he rose to the highest academic positions, he never ceased to be a field scientist. Moreover, like Émile Brumpt in Central Africa and Camille Desportes in West Africa, he led his collaborators far from the laboratory benches, collections and libraries to remote dangerous bivouacs where discoveries are always possible. His research surveys took him to Morocco (1949), Iran (1951), Madagascar (1957), the Central African Republic (1965), Côte d’Ivoire (1966), French Guiana (1970), Brazil (1972), Guadeloupe (1973), Malaysia (1974) and Australia (1976). The “Chabaud team” always came back full-handed from these missions with abundant study material and ideas for innovative projects. Moreover, on these occasions, several renowned foreign scientists – such as Ian Beveridge and Dave Spratt – were regular collaborators and even became French at heart! This epistemological brilliance gave rise to an exceptional parasitology centre at the *Muséum National d’Histoire Naturelle* (MNHN) in Paris. This “scientific beehive” bustled day and night with researchers coming and going, each focussed on identifying and describing an unknown “entity”, revising a prestigious collection or acclimatising a parasite reservoir or vector. The successful installation of a new parasite was always joyously celebrated – a previously enigmatic cycle would finally reveal its secrets!

In this spirit, in June 1968, the entire Chabaud team fled the “Parisian shambles” to take refuge in the French Montagne Noire Orientale region, setting up shop in the abandoned village of La Borie Nouvelle (Hérault department), where our Montpellier Leishmaniasis Ecology team camped each summer to study the trophic behaviour and natural infestation patterns of Phlebotominae sandflies. The “Parisians” brought their equipment and optical instruments to carry out their own studies or to finish writing a scientific PhD thesis. At nightfall, however, most of them, led by their “boss”, joined our group to participate in determining the circadian cycles of vectors or isolating *Leishmania*. These special moments enabled us to perfect our own techniques, but also gave rise to many unique ideas on the structure, function and specificity of the sandfly-*Leishmania* system.

Moreover, both in the laboratory and field, multidisciplinarity was an operational imperative for Chabaud. Here I should mention the late Francis Petter, Zoology Professor at the MNHN and recognised expert on desert rodents, especially Gerbillidae. Francis Petter knew better than anyone how to achieve successful catches, transport the vigorous captured animals and ultimately install them in the Parisian animal laboratory. Before him, the continuous reproduction of some parasites was considered unlikely. However, after his intervention, several new worm and protist taxa, along with their vectors and reservoirs, were successfully reproduced in parasitology and pharmacology centres worldwide.

### A visionary teacher-researcher

Upon receiving his Doctor of Medicine degree in 1945, Alain Chabaud was employed by the Parasitology Service at the Faculty of Medicine in Paris, headed by Émile Brumpt. After serving as a preparation officer, he was appointed as Assistant (1947), Project Leader (1948), and finally Senior Associate Lecturer (1952). He also continued his studies at the Faculty of Science, where he obtained a Bachelor of Science (1947) and a PhD in Natural Science (1954). Meanwhile (1950), he became Deputy Director at the *École Pratique des Hautes Études* (EPHE) in the Helminthology and Comparative Parasitology laboratory founded by Robert-Philippe Dollfus. He became Director in 1958. Two years later, at the age of 37, he was appointed Professor at the MNHN. A traditional Chair (zoology, helminthology, in French *Zoologie, Vers*) was specially created for this purpose, which became an efficient and flexible structure that he continued to manage, even after his retirement (1990). Of course, this early success was related to his fine intelligence and strong will but, as he pointed out when he was awarded a silver medal by the *Société Française de Parasitologie*, he was also active during the “30 glorious years” after the war, when French research achieved the highest level of excellence. Large research organisations such as the CNRS (1939), INRA (1946) and INSERM (1964) also emerged during these dynamic times. It was an economically prosperous period which enabled the creation of excellent well-equipped laboratories with high-performance instruments, as well as motivated researchers and technicians. These were headed by “real bosses”, in the conventional sense, with charismatic Alain Chabaud being one of them.

### Promoter of the evolutionary paradigm

Seldom is a *Review of Titles and Research* (in French “*Épreuve de Titres et Travaux*”) fully representative of a researcher’s career, and this is the case with Alain Chabaud. With a clear and direct writing style, in two issues, he provides an overview of his teacher-researcher career. In each one, he presents the results obtained in a key period of his professional life. When reading them, I was overwhelmed by an intense jubilation, that of my youth as a researcher when I was being trained by the naturalists Hervé Harant and Louis Emberger in Montpellier.

The first issue (1960) traces the integration of the doctor and naturalist Alain Chabaud into the Parasitology laboratory at the Medical School in Paris (1946–1960). His scientific precocity was admired by all, for two reasons. First, the general nature of the courses and laboratory work, dealing equally with fundamental parasitology, epidemiology and exotic pathology. This versatility led him to focus research throughout his career on many groups, including parasites, vectors, arthropods, molluscs and vertebrate reservoirs. Second, the diversity and expertise of the teachers he was in contact with daily. This included Jacques Callot, Camille Desportes, Robert-Philippe Dollfus and Maurice Langeron, not to mention foreign scholars who came to work with Brumpt’s team for further inspiration. Two of the most prestigious of these were Professors P.C.C. Garnham and Ettore Biocca.

The *Review of Titles and Research* begins with a tribute to the head of the service Émile Brumpt, but for us the interest lies less in such conventional praise and more in the presentation of new evolutionary parasitology paradigms. These were initially based on nematodes, a complex group, but which allowed Alain Chabaud to establish close links between certain highly evolutionary traits and the corresponding larval or sexual stages. The evolutionary line, host-parasite coevolution and capture concepts were among the fundamental upshots, not to mention the fascinating linear or punctual speciation issue. In all projects, the teacher asked his students to take the host ecology and chorology into account, along with the intrinsic plasticity of the parasites – which was later followed up by molecular geneticists. His enthusiasm for the continental drift concept should also be mentioned, which was a revolutionary theory at the time and brilliantly promoted by Max Vachon, his colleague at the MNHN.

The second issue of the *Épreuve* (1977) reviewed the results obtained after practical application of the paradigms and new concepts. It reveals the second scientific life of Alain Chabaud, as a Professor at the MNHN and respected school head. He was officially invited to many countries worldwide, including: Madagascar (Institut Pasteur, 1961), Italy (First International Parasitology Conference, 1964), the Central African Republic (La Maboké research station, 1965), Congo-Brazzaville (ORSTOM, 1966), Iran (International Tropical Medicine Conference, 1968), Czechoslovakia (Parasite Ecology Conference, 1970), Canada (University of Guelph, 1970), the USA (Second International Parasitology Conference, 1970), the Soviet Union (USSR Academy of Science, 1973), Austria (Third International Parasitology Conference, 1973) and Brazil (France-Brazil Cooperation, 1976). In 1968, the *Institut de France* awarded him the *Prix Foulon*. A year later he was elected President of the French Zoological Society. Moreover, although Alain Chabaud was not particularly attracted to honours, with great spontaneity and tact he accepted to be decorated with the rank of Chevalier in the French National Order of the Legion of Honour (1974). This distinction was especially dear to him, especially as the request was strongly supported by his colleague and friend Jean Dorst, Zoology Professor, MNHN Director and tireless advocate of wildlife, flora and living environments.

### Research work

To keep this tribute light, we pooled together a few examples of Alain Chabaud’s work. Although the first studies were conducted at the Faculty of Medicine, most – especially those at the MNHN – were responses to tenders from universities, the CNRS, WHO, INRA and MNHN. Most of these studies included external collaborators and enrolled students. Moreover, in the spirit of our late colleague, we will comply with the systematic order followed in the *Review of Titles*, since systematics is the steadfast basis of parasitology, including genetics, epidemiology, biogeography, ecology and evolution.

#### Nematodes

Parasite taxonomy was thriving in the 1950s. Until then, phylogenetic traits were barely utilised and the discipline lacked new models. From the outset, Alain Chabaud saw the potential of nematodes for solving some of these problems, especially as this highly morphologically and ecoepidemiologically diversified group has a major free component that facilitates studies to trace the history back to the common ancestors.

At the Faculty of Medicine in Paris, Alain Chabaud was already surrounded by competent and efficient students such as Annie Petter, specialist of nematodes of fish, amphibians and reptiles, and Yves Golvan, specialist of Acanthocephala. At the MNHN, other equally outstanding collaborators joined them, including Odile Bain, Marie-Claude Desset, Jean-Pierre Hugot, Jean-Claude Quentin and Jean-Lou Justine.

In this regard, we will devote a few lines to strongyles, a complex group that was studied especially by Marie-Claude Desset, currently Emeritus Research Director at the CNRS. We will consider two discriminating characteristics, i.e. “bursal ribs” (in French *côtes bursales*) and the “synlophe”. (1) Bursal ribs, which form the backbone of the caudal bursa in *Strongylus* species, are considered to be homologous to cloacal papillae in free rhabditid nematodes. Hence, these parasitic nematodes could be derived from a common Rhabditidae-Stronglidae ancestor, which probably appeared in the early Tertiary. (2) The neologism “*synlophe*” was coined by Robert-Philippe Dollfus to designate a longitudinal cuticular formation with ridges that enable the worms to attach to the host’s intestinal villi. Studies on this revealed most of the inventoried systematic categories and the evolutionary significance. Moreover, results obtained with adult strongyles could be applied to larvae infecting the L4 stage. The famous and often criticised aphorism of Ernst Heckel, “*ontogenesis recapitulates phylogeny*”, found a new justification in parasitology. A few years later, thanks to Pierre Darlu’s intervention, the application of cladistic techniques enabled phylogenetic reconstruction of the superfamily Trichostrongyloidea. Its monophyly was confirmed, along with its diversification into three branches, corresponding to the three families already differentiated by traditional systematics. Finally, the overall results indicated that Ratite flightless birds were ancestral hosts of this group. These results were further confirmed when molecular characteristics were applied to the suborder Trichostrongylina.

The continental drift theory was also applied in this research, as demonstrated in the following example. From the beginning of their research, the Chabaud team took natural traits into account and the findings suggested that the subfamily Heligmonellinae, i.e. parasites of African Phiomorpha rodents, was older than Pudicinae, parasites of Caviomorpha rodents. In other words, the individualisation of Caviomorpha rodents could have given rise to Pudicinae. However, this strictly parasitology-based conclusion could be extended much further – it supported the readily criticised hypothesis of the paleontologist-tectonics specialists René Lavocat and René Hoffstetter. For these authors, African-American continental drift gave rise to the American Caviomorpha line from African Phyomorpha. Thereafter, the low ecological pressure that supposedly prevailed in the newly colonised American territory could explain the Caviomorpha genus-species diversification that took place. The phyletic considerations of nematologues thus supported the hypothesis of the transformative migration of the mammal hosts. The host-parasite coevolution (“microevolution”) process could thus explain the Pudicinae diversification that took place.

In Epidemiology, “capture” corresponds to the establishment of a pathogenic organism in a new host, systematically and geographically remote from the original host. This phenomenon, which was already known in parasitology, was considered simply as a cycle accident and, for some researchers, had no major impact on the evolution of the parasite. With his students, Alain Chabaud demonstrated that, conversely, the phenomenon was crucial in many nematodes and could have been due to a nonspecific host feeding behaviour. The presence of the same strongyle species in ruminants and lagomorphs was due to a common food source, i.e. grasslands, thus providing a wealth of information for veterinarians. In other cases, further back in history, capture was the cause of major changes (“macroevolution”), for instance in the Molineinae, a group represented in both amphibians and ruminants. This transfer probably occurred in the early Eocene.

Another important topic, filariae, was studied by Alain Chabaud and several of his students, including Odile Bain. Emeritus Research Director at the CNRS, Odile Bain devoted most of her career to the systematics, epidemiological cycle and physiopathology of over 20 filaria species. Her studies on the behaviour of microfilarial worms in the digestive tract of vectors are models of excellence. Unlike traditional filaria specialists, she used a so-called “comparative” approach that was dear to Émile Brumpt’s school. Through many missions, she analysed the systematic and biological diversity of the group. She described several new species in mammals, reptiles and amphibians. Some gave rise to formal models of major interest in fundamental pathophysiology (“parasite specificity”, “facilitation, limitation and proportionality” phenomena). Continually considered, several of them were further developed in French and foreign parasitology and pharmacology laboratories. In this regard, we should mention Jean-Claude Quentin’s efficiency in establishing experimental cycles.

Like the previous topic, oxyurids were studied by Alain Chabaud early in his career. First, in collaboration with Ettore Biocca and Yves Golvan, studies of Moroccan, Senegalese and Somalian oxyurids, and then with Jean-Pierre Hugot on the phylogenetics of the group overall. The later carried out phylogenetic taxonomy research at a time when informatics were just emerging, and considered that oxyurids could be ranked as true evolutionary markers.

#### Trematodes

Alain Chabaud’s interest in the “chaetotaxy” of trematodes was confirmed by Josette Richard, Christiane Dufour and Jean-Louis Albaret. We owe this success to the transfer, by Jean-Louis Albaret, of Pierre de Puytorac’s elegant silver impregnation technique. This technique, when applied to cercaria and miracidium species, helped reveal an ancestral sensillar structure. Considering the advantages of this method, several external laboratories (Montpellier, Perpignan, Marseille, Lille), with a broad range of sophisticated equipment, collaborated closely on the project.

Finally, we should point out the effective participation of the MNHN team in the DGRST “*Ecology of Schistosomiasis in Guadeloupe*” concerted initiative. For 10 years, ecoepidemiologists compared their hypotheses with field findings. The fact that the disease is now eradicated is evidence of the quality of their research.

In addition to this research, the systematics and ethology of free Platyhelminthes (Turbellaria) and parasites were substantially developed by Jean-Lou Justine.

#### Haemosporidia

Studies on the systematics and biology of *Plasmodium* – a priority topic – was placed under the responsibility of Irène Landau, MNHN Professor, currently one of the leading specialists of the group.

Like the strategy used with strongyles, which was dear to him, Alain Chabaud recommended a comparative approach to *Plasmodium*. Based on the observation of many haemosporidian cycles in reptiles, birds and mammals, Alain Chabaud and Irène Landau hypothesised that, in *Plasmodium*, gametocytes and the early stages of the exoerythrocytic cycle appeared before blood stage schizogony. A few years later, they highlighted two forms of hepatic schizonts in several rodents: one large-sized and thin-walled with many nuclei, and the other smaller and thick-walled with rare scattered nuclei. The first schizonts rapidly disappear from the liver, while the latter remain until the host’s death. These subquiescent forms explain the many continuous relapses, while also representing real natural *Plasmodium* maintainers.

This scholarly inventory work, carried out during long-term missions, led the authors to discover many new species. These included the emblematic *P. yoelii* and *P. chabaudi,* present in the arboreal rodent *Thamnomys rutilans.* Strains of these two *Plasmodium* species, which are easily maintained in laboratory mice via laboratory-reared *Anopheles stephensi*, could be disseminated worldwide. They are the source of hundreds of publications and still used to carry out many pharmacology studies.

### Founding of the French Society of Parasitology

As an authentic school head, Alain Chabaud certainly knew how to convince the most voluntary and brightest researchers with his ideas, followed by their administrative and social promotion. This same efficiency could be noted in his university activities. The founding of the *Société Française de Parasitologie* (SFP) was significant in this respect.

In 1962, the World Federation of Parasitology Societies prepared its first international conference, which was held in Rome under the chairmanship of Ettore Biocca. At the same time, Alain Chabaud wanted French parasitology to take its rightful place in the scholarly community. He thus took advantage of the meeting in Rome. In Paris, he mobilised the most motivated of us, including Jean Biguet, Jacques Callot, Jean-Marie Doby, Claude Dupuis and Pierre-Paul Grassé. The SFP was founded on 7 April 1962 based on a decision of the Constituent Assembly that met at the MNHN Parasitology Laboratory. Claude Dupuis was asked to oversee the interim Secretariat, soon followed by Alain Chabaud, until 1975. Since its launch, the SFP has continued to be the backbone of our community – a multidisciplinary structure that remains devoted to its founding principle, i.e. theoretical and applied study of the “parasitic fact” (or “parasitism”). Its success was the result of the close collaboration of several teams using complementary approaches, such as taxonomy, phylogeny, biogeography, ecology, genetics, epidemiology and physiopathology, in both medical and veterinary fields. Note that large research organisations (the CNRS, INSERM, INRA, ORSTOM [now IRD], CIRAD, IFREMER), universities of Science and Pharmacy and university hospital centres (CHU) have tirelessly supported this activity.

### The “*Annales de Parasitologie humaine et comparée*” (now “*Parasite*”)

Alain Chabaud considered it his moral duty to support the “*Annales de Parasitologie humaine et comparée*”, a prestigious publication that was founded by Émile Brumpt, Maurice Langeron and Maurice Neveu-Lemaire in 1923. For many years, he was the “soul” of this publication, always attentive to its financial equilibrium, and the quality and timeliness of the publications. Being a MNHN Professor, this task was relatively easy, especially since he was also responsible for publishing the “*Mémoires du Muséum National d’Histoire Naturelle*”. However, despite his efforts, *Annales* had to be purchased from the publisher Masson. At this time, the decisive intervention of René Houin helped preserve the spirit of the publication. Only the title changed, from “*Annales*” to “*Parasite*”. The journal *Parasite* recently went online thanks to the dynamic involvement of Jean-Lou Justine, another brilliant collaborator of Alain Chabaud.

### In farewell

On 13 March 2013, an exceptional parasitologist, Alain Gabriel Chabaud, left us just as he was turning 90 years old. With a humanistic temperament, a trained teacher-researcher, throughout his professional and personal life he was a model of competence and grace, as highlighted by the strength of his ideas, the value of his publications and the loyalty of his colleagues and students. All sectors of the scientific community paid him a vibrant tribute, claiming that he was “the best of all of us”. In turn, and with great pride, I honour this great pathfinder. I express this, not only as former President of the French Parasitology Society, but also as a friend and follower.

## A personal Hommage

by Marie-Claude Durette-Desset, with contributions from Jean-Louis Albaret, Christiane Bayssade-Dufour, Jimmy Cassone, Irène Landau, Annie Petter and Roselyne Tchéprakoff

This obituary has been prepared together with contributions from a number of colleagues and others associated with our laboratory, in order to include our joint thoughts concerning Professor Chabaud. I take this opportunity to thank them all for permitting me to select from their manuscripts those points that seemed to me to be the most relevant in describing his personality.

My teacher, Professor Chabaud, whom we addressed simply as “Chabaud” using the French familial form of “tutoyer”, departed this world at the age of 90, having made a profound impression for more than 50 years in the field of parasitology, both in France and at the international level.

Having qualified both in Medicine and as a Doctor of Natural Sciences, he joined the Museum at the age of 38 as the first Director of a Chair that was newly created for him and which he named the Laboratoire de Zoologie (Vers). Originally he had wished to enter the Ecole navale to follow in his father's footsteps, but was not accepted because of his poor eyesight (myopia). This proved to be an unanticipated opportunity for the discipline of parasitology in France.

From their initial encounter he would greet newcomers with outstanding warmth. Many individuals, some of whom were later to become research workers, technicians or administrators, expected to feel intimidated by their initial encounters with a Professor of the Museum. In fact, they were received with remarkable informality and kindness by a very young director, constantly shadowed by his three-legged dog!

This informality was a constant feature of his life. He was never behind in participating in collective tasks; for example, the archiving of the historic collections of Professor Dollfus. How many times he assisted in feeding the infected rodents in the animal house! How often he donned his white lab coat to help with dissecting mosquito salivary glands!

This simplicity of behaviour was associated with an outstanding modesty. Chabaud had a horror of being put in the forefront and detested medals and other honours. Therefore, even though it was a standard tradition in the Museum for new appointees to give an inaugural lecture (Professors being obliged to participate in teaching), Chabaud only agreed when the Museum threatened that unless he did so he would not receive his salary! Years later the Director of the Museum expressed a wish that every Professor should be made a member of the Légion d'honneur. Chabaud opposed this idea. He had to agree, however, but the ceremony in his case took place in the kitchen washing-up room of the rue Cuvier in the presence only of his wife and his students. He accepted to be made a recipient of the Emile Brumpt Prize, but only jointly with Irène Landau. Finally, he was nominated to receive the silver medal of the French Society of Parasitology at a session of the European Society of Parasitology. When some time later I expressed surprise to Renée Houin that Chabaud never received the gold medal, he told me that, because of the limited finances of the Society, the medal never came into being!

Chabaud and his dog were inseparable and it accompanied him everywhere. On the whole the laboratory staff rapidly adapted to this situation even though sometimes they did not think too highly of it. I remember that on one occasion a visitor to the lab was lightly bitten on the leg, but fortunately did not complain. I also recall the occasion when one of our colleagues gave the dog a few kicks under the table during a communal meal. Fortunately, neither that bitch nor a succession of Chabaud’s later animals ever protested!

On another occasion, together with his friend and collaborator Charles Berger we all went to the cinema. Barka the three-legged Alsatian came with us, of course. Chabaud had put a scarf on her head and carried her in his arms. The cashier let them pass and everything went well until some dogs appeared on the screen and started to bark and Barka decided to reply. Amidst our laughter we were chucked out without consideration.

A short distance behind dogs, Chabaud adored small children, with whom he also insisted on tutoieing. He let himself be led around on the ground by his tie (which in those days he wore!) by a 4-year-old François Petter. He presented my 8-year-old daughter with cheap perfumes which she was mad about, which infuriated me. At table he always took the infants' side when a parent ticked them off or tried to make them finish up their plates. He took advantage of their presence to get up to all sorts of mischief.

We always had the right to bring the children to the lab when they had time off from school. He organised the daily timetable for those among us who had young children. He had a high regard for everyone and gave his staff a great deal of attention. He took great care when filling in applications for promotion submitted by his technicians. Always gallant, he never allowed the cleaning lady to carry a heavy water container through the long corridor of the rue Cuvier when he saw her. In spite of current ideas on social behaviour, this kind of action in no way reduced his authoritative position nor prevented him from chastising people, even if this meant holding back his temper even when he was displeased.

Here one must mention the story of the laboratory van which conveyed us for over a dozen years to various congresses and meetings of the French Society of Parasitology, and which also saw service on missions to the south of France and to Corsica. This emblematic van ended its career in 1981 when the Museum decided that its job should include moving us from the rue Cuvier to the rue de Buffon to a larger laboratory. The male members of staff (Chabaud included) carried out the move as inadequate support was provided by the Museum. Chabaud decided to provide the new lab with furniture purchased from Emmaüs and in this way the cost of the move could be met. Room was found for certain items, such as the famous “Cuvier buffet” as named by Chabaud because of the animals that had sculpted it, in the room which was officially named the “washroom” since it was there that the glassware was cleaned. Of special importance were the glasses that permitted us to gather together at midday for lunch or in the afternoon for tea which, at times, became transformed into wine. The luncheon courses were prepared by volunteers of whom Chabaud was often a member. Visitors in transit through the lab were always invited by him to join us at lunch, and he used to say that the time they joined us for a meal did more to improve international relations than congresses. Moreover, most of the visiting parasitologists became our friends.

Another place that in a sense served as an extension of the laboratory in Paris was La Bunelière, which we referred to as “The Buno”. Chabaud’s country house was open to all and there, too, we alternated between work and walking the dogs. In the course of these walks Chabaud, true naturalist that he was, pointed out the names of all the plants or identified them with the help of Odile Bain. Moreover, he had an extensive knowledge of insects and had a magnificent collection of butterflies. Finally, at the end of the mushroom season, thanks to Nicole Léger who taught us to identify them, we passed unforgettable evenings cooking our collections while chatting about this and that, but mostly about science. And all this in a youthful, relaxed atmosphere, frequently in the company of his French or foreign visitors.

These informal occasions in no way lessened the value of a fruitful and extensive period of research and teaching. An unquestionable authority underlay his youthful appearance. In the single year of 1971 four individuals successfully presented national University theses; the number of works published by his teams after the creation of a Department of Protozoology rose to about 40 per year. Moreover, the new department included over a period of 30 years’ research workers from all over the world who joined in the production of high standard diplomas or professional qualifications.

I hope that some of these observations will recall happy memories to those who knew Chabaud and provide a picture for those who did not (albeit limited because of a shortage of time) of his so rich and complex personality. He demanded a lot from us but set himself as our example. He gave us a taste for good, careful work and honesty in our scientific output. He taught us how not to take ourselves too seriously, but always to do so with our work. He knew how to show technicians how interesting and important their work was and to be proud of their contributions to the national scientific collections. He showed the research staff how work can become a pleasure. He also pushed every one of us to his or her intellectual limits and many, myself especially, could never have become devoted to a life in international research without his constant advice and personal attention.

Chabaud, thanks to his brilliant intellect, his editing skills, his passion for the natural sciences, his dynamic and happy personality, his prodigious memory and his great charisma will, in addition to the deep and unforgettable personal impression he made on all those who met him, have contributed in a masterly fashion with the massive influence he exerted on the discipline of parasitology, both internationally and in France.

Translated by W. Peters

